# Art therapy and brain injury: making the invisible visible

**DOI:** 10.3389/fpsyg.2024.1489813

**Published:** 2024-12-04

**Authors:** Denise R. Wolf, Michele D. Rattigan

**Affiliations:** College of Nursing and Health Professions, Art Therapy and Counseling, Drexel University, Philadelphia, PA, United States

**Keywords:** traumatic brain injury, post-concussive syndrome, art therapy, imagery, expression, interprofessional collaboration, translation neurosciences

## Abstract

The multiple cognitive, somatic, and behavioral changes following head injuries can result in expressive language difficulties that may not be resolved quickly. This paper explores the traumatic brain injury and post-concussive syndrome artwork created by an art therapist and the child of an art therapist, making the invisible neurological consequences of head injuries visible. Our first-person and caregiver perspectives offer examples of visual arts-based communication between patients, health professionals, and family members. Utilizing client imagery as a form of communication may improve patient outcomes through the identification and resultant treatment of overlooked and underdiagnosed symptoms. Experiences such as confusion, fear, localized pain, and mood lability stem not only from the injury itself, but from the experience of damaged microstructures that are often undetectable in standard diagnostic testing. Additionally, symptoms such as temperature and appetite dysregulation, vestibular and proprioceptive disruptions, and circadian rhythm sleep disorders evade standard diagnostic inventories. This may prompt the patient to question the reality of their somatic and cognitive experiences. Research supports the position of the authors: these experiences can be communicated through client imagery, expediting healing and improving overall health. In the spontaneously created art traversing child and adult stages of development, we discovered multiple prevalent themes within the imagery too numerous to ignore. Practice recommendations will be discussed for both art therapists and interprofessional healthcare collaborators concerning the use of imagery and visual expression when working with those who have sustained traumatic brain injuries.

## Invisible damage, underdiagnosed experiences, and implicit bias

“But there are certain meanings that are lost forever the moment they are explained in words” (Murakami, 2011, p. 583).

Over 1.7 million traumatic brain injuries (TBIs) occur each year in the United States across all age groups (Georges and Das, 2023), with mild TBI (mTBI) comprising over 90% of cases presenting to hospitals (Maas et al., 2022). Thirty to 80% of reported cases will experience post concussive syndrome (PCS) symptoms, and likely live with longer-term challenges (Georges and Das, 2023; Goldsmith, 2024; Schwab et al., 2021). Resultant cognitive, behavioral, and/or personality changes can be significant (Trevena and Cameron, 2011; National Academies of Sciences, Engineering, and Medicine, 2022). These statistics are underestimated as they do not account for misdiagnosis and those who did not seek medical attention (Fried et al., 2022; Selassie et al., 2008; Setnik and Bazarian, 2007; Thurman et al., 1999; Zaloshnja et al., 2008). A barrier to symptom identification is the compromised brain’s ability to engage in verbal communication and expression (Walker et al., 2016).

Visual art products can fill communication gaps as effective tools between patients, caretakers, and healthcare providers (Lydia and Aurora, 2014). Art therapy, specifically, can document mTBI symptomatology and help survivors articulate their experiences, making the invisible visible; as the artwork created in art therapy documents and makes tangible the mind–body connection (Hass-Cohen, 2008; Kemp and Gregory, 2024). By synthesizing the multiple lenses of patient and caregiver first- and second-person accounts of mTBI and PSC, our experiences as art therapists and art therapy educators, and contemporary literature, this perspective paper aims to advance the diagnosis and treatment of acquired brain injury.

We also acknowledge the implicit bias surrounding gender and race that further compounds TBI diagnosis and treatment. Health care disparities impact racial and ethnic minoritized groups, the uninsured, and those in rural areas (ASHA, 2013). As an example, a colleague of ours sustained minor external injuries in a car accident. Several days later, she began having unprecedented seizures and was taken back to the hospital. After examination, the medical team determined the seizures were due to her feeling unappreciated and working too hard. The doctor’s suggested course of treatment was for her husband to “buy her jewelry;” she would feel appreciated, and the seizures would subside. Our colleague’s case is one instance, yet treatment outcome research *is* lacking for females, older adults, and people from low income and middle-income countries (Maas et al., 2022). For both children and adults, changes in behavior and affect regulation may lead some physicians to attribute the majority of mTBI symptoms to psychological causes *only*, which can also lead mTBI symptoms to be similarly disregarded by the person affected, their family, and caregivers (ASHA, 2013; Scaer, 2001).

Depression and anxiety are common complications after sustaining a mTBI and dissipate later in recovery (Zwilling et al., 2022), recognizing that “recovery” can also denote years following the onset of injury. In instances of co-occurring post-traumatic stress disorder (PTSD) and TBI, recovery time could mean decades after injury (Jones et al., 2018). Guillamondegui et al. (2011) completed a systemic review for the Agency for Healthcare and Research Quality of 112 publications investigating the relationship between depression and TBI: Its prevalence, best-practice screening tools, co-existing psychiatric diagnoses, and treatment outcomes of depression following TBI. It was noted that while generalized anxiety was the most common comorbid diagnosis with depression and TBI noted in the literature, PTSD was second; sometimes included in the screening tools for anxiety and not with a diagnostic tool specific to PTSD (Guillamondegui et al., 2011). The AHRQ authors’ summary criticized the state of treatment for TBI and depression experiencers in the U.S. at that time, specifically that literature was lacking in evidence-based treatment options for the growing population of people affected by depression due to TBI (Guillamondegui et al., 2011). To further demonstrate how emotion, misdiagnosis, and miscommunication can occur in the detection and treatment of mTBI, we will share our first- and second-person accounts. Correspondingly, we will share how artmaking became a bridge for intra- and interpersonal connection (Hass-Cohen, 2008), expression, and understanding for an adult and child with PCS and mTBI; and how this helped both authors fortify their understanding of the art therapy - neuroscience connection.

### Michele’s story

**Figure 1:**
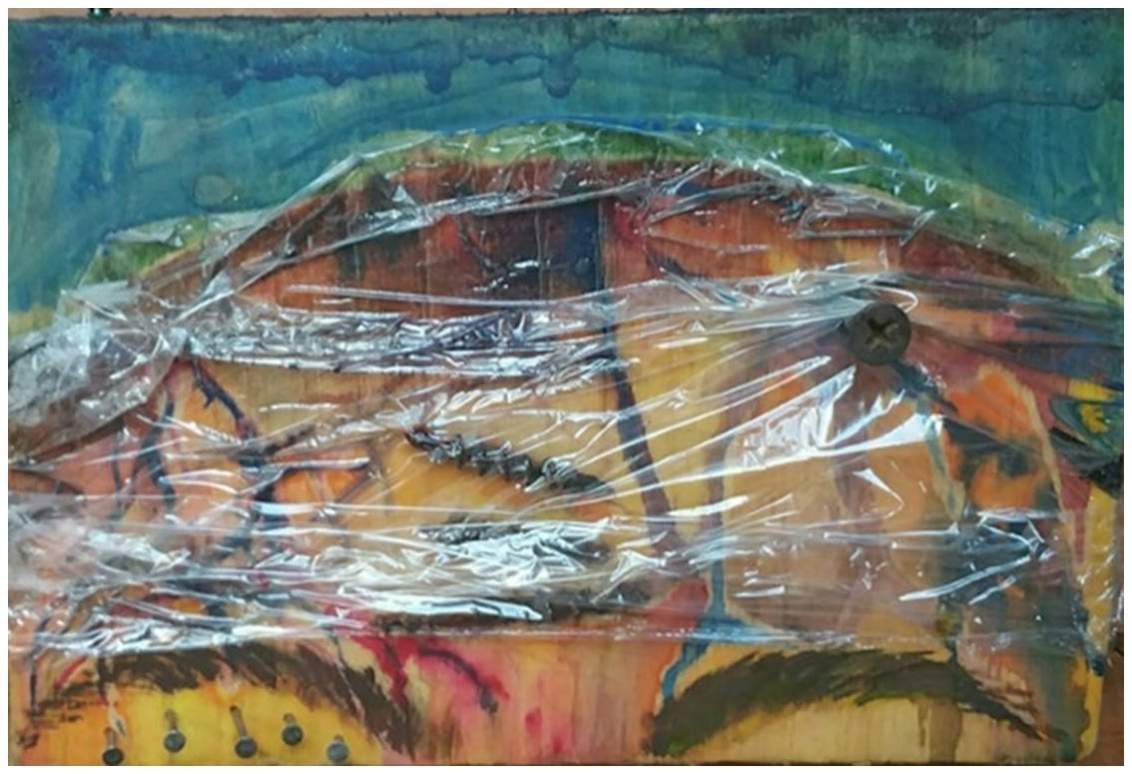
Michele’s sculpture 6 months after the accident. Close-up (detail) of adult art used to describe their symptoms to their healthcare provider.

A day after the minor car accident I was checked for neck pain and a severe headache, was told to go home, have a glass of wine, and relax because I’d “be fine.” Six months later I handed a different doctor a small, painted block of wood wrapped in cellophane with large black nails sticking out of it because I could not describe how sharp and intense the headaches still were (Figure 1). I worried no one believed me. On the outside, I looked “normal,” but I was struggling. I could not focus, my ears were ringing, the lights were too bright, the sounds were too loud, and I was exhausted. This current physician told me I had sustained a mTBI after the accident and now had PCS and benign paroxysmal positional vertigo. Two years of medications, vestibular physical therapy, restorative yoga, acupuncture, and my own art brought me out of that scary, confusing, and painful place. All these were necessary to instill wellness and healing. Medication for symptom management, vestibular physical therapy to help the brain better adapt to impaired signals coming from damaged areas, and yoga and acupuncture to relax areas of muscle strain and reduce stress are viable treatments. However, people may not consider how the art therapy process and product can be essential treatment tools in brain injury care.

As Michele’s story introduced, it is important to address both the cognitive and somatic impact when people’s pain and symptoms are minimized and/or underdiagnosed. This makes for a challenging experience as mTBI survivors are already doubting the reality of what they feel (Ramos-Zúñiga et al., 2014). It can be just as difficult for doctors and other healthcare providers; mTBI and PCS are like the junk-drawer in your kitchen filled with “non-specific symptom clusters” (Clark et al., 2022, p. 1906) representing layers of past and current comorbid injuries mixed with other factors. For instance, the TBI-initiated senescence-associated secretory phenotypes indicating cell arrest and inflammation cause cellular damage (Marya and Patel, 2021; Tominaga et al., 2019), are exacerbated by stress, oppression, toxic environments, and trauma (Marya and Patel, 2021) and can worsen over time due to damaged DNA (Hiploylee et al., 2017; Schwab et al., 2021). I, Michele, certainly felt stuck as both a PSC experiencer and a healthcare provider, questioning my temporary disabilities. I felt frozen in a thick, wavy glass box unable to connect. Perhaps my care providers felt similarly trapped, assessing what was “real” and what was psychosomatic. In hindsight, it was all real; including the way I was initially dismissed and misdiagnosed.

### Non-specific symptoms

Common symptoms of mTBI present as physical, visual, auditory, vestibular, neurobehavioral, and/or cognitive-communicative (Ryu et al., 2009; ASHA, 2013; Goldsmith, 2024;). Diagnosis is formulated based on patient self-report post-injury, often after enduring symptoms for weeks or months. The outward signs are invisible; the person may appear physically well but may feel and act otherwise (ASHA, 2013). Symptom identification is notoriously elusive, as mTBIs lack obvious signs of brain trauma in neuroimaging (Padula, 2016). Adverse impacts on one’s overall wellbeing include significant deficits in cognitive, behavioral, and/or personality domains, continuing for some time following injury (Trevena and Cameron, 2011) including resultant PCS. Post-concussive syndrome includes subjective somatic, cognitive, and emotional problems such as headache, dizziness, forgetfulness, inattention, and depressed or anxious mood that present 12 months or more after injury (Kline, 2016; Mayo Clinic, 2023; McNally et al., 2013). Highly debated amongst medical professionals and researchers, PCS prevalence rates can vary between 11 and 82% (Polinder et al., 2018).

Children experiencing TBI symptoms often present with headache, fatigue, inattention, forgetfulness, slowed cognitive processing, irritability, and disinhibition (Trevena and Cameron, 2011; Yeates, 2010). Even though TBI is the leading cause of death and disability in children in the US (Ferrazzano et al., 2024), there are reportedly few studies that document the pervasiveness of the symptoms (McNally et al., 2013; Yeates, 2010). Adults may experience an alteration of consciousness, slowed information processing, short-term memory impairment, and poor concentration. They may additionally report severe headaches, pervasive fatigue (cognitive and physical), balance difficulties, tinnitus, feelings of detachment, anxiety, irritability, apathy, and depression (Scaer, 2001).

### Denise and Rheva’s story

“Mommy, the ground is not lumpy, right?” My 9-year-old daughter inquired with a pleading look in her eyes. We were visiting the library for the first time since the accident a few months prior when she was struck by a car while crossing the street. “No, it’s not,” I replied, “but it makes sense that you are seeing and feeling that.” If I was not a trauma therapist with an understanding of basic neurobiology, I may have been alarmed by this question. For months after the initial impact, I had fielded reality-oriented questions from her so she could see if her experiences of the world, her body, and her cognitive processes, fit the facts. Ranging from changes in altered hypothalamic functional connectivity (Lu et al., 2020) like temperature regulation and appetite reduction, to ongoing changes in vision and sensitivity to light and noise (CDC, 2024), my daughter’s ‘non-critical’ symptoms were largely ignored by doctors and specialists. Although the initial treatment addressed life-threatening injuries like her punctured lung and lacerated liver, nurses and doctors ignored my repeated requests to attend other symptom presentation. I observed that her left eye seemed to roll around without partnership with her right eye. Strabismus, crossed or misaligned eyes, is a common resulting condition of brain injury. Ten years later, she remains sensitive to sound, light, and proprioceptive input. Contemporary literature calls for both ocular motor impairment (McDonald et al., 2022) and auditory disturbance (Šarkić et al., 2023) to be explored as potential objective biomarkers to help in the diagnosis of mTBI, however we experienced continual disregard of “secondary symptoms.”

### Visual imagery as a diagnostic tool

Art has been called symbolic speech (Naumburg, 1987) and soul language (Wilson, 2003). Imagery usage is a specific advantage in art therapy because we think first in images (Wadeson, 2010). Translation of our thoughts-to-art are streamlined unlike thoughts-to-words (Rattigan, 2021; Wadeson, 2010). For TBI patients, art gives voice to their experiences (Kemp and Gregory, 2024; Spectrum Health Newsroom, 2024). To help discern patients’ experiences, studies have linked visual symbols with existing clinical assessment tools (Cohut, 2018). The non-verbal component of art therapy utilizes brain processes that do not depend solely on expressive and receptive language (Rankin, 2008). “One of the most direct ways that neuroscience can be applied to art therapy is in the validation and normalization of emotional states and experiences” (King et al., 2019, p. 151). Patients’ visual symbols are individual expressions; however, they can also identify unique neurological impairments and establish common patterns across the lifespan and cultures (Cheyne-King, 1990). Patient artwork can also significantly aid assessment and treatment (Walker et al., 2016) for art therapy and non-art therapy providers. In the treatment of concussion-related illness, a collaborative, interprofessional approach to diagnosis and treatment is key (Polinder et al., 2018; Walker et al., 2016).

### Common visual themes across the lifespan of mTBI patients

Michele took it upon herself to make art daily with a black ink pen in 3-inch by 5-inch journals to document the ups and downs of her recovery. Michele is an artist and an art therapist, but art journaling is not restricted to artists, and artmaking for expression and communication does not require art education, skill, or talent. In fact, contemporary studies have found that the cortisol levels of adults, regardless of skill level, media selection, or gender, reduces after open-ended art making (Kaimal et al., 2016); and further, the democratizing effect of doodling in comparison to other types of structured drawing prompts initiate the highest measured blood flow in the brain’s reward pathway via a functional near-infrared spectroscopy (Kaimal et al., 2017).

**Figure 2:**
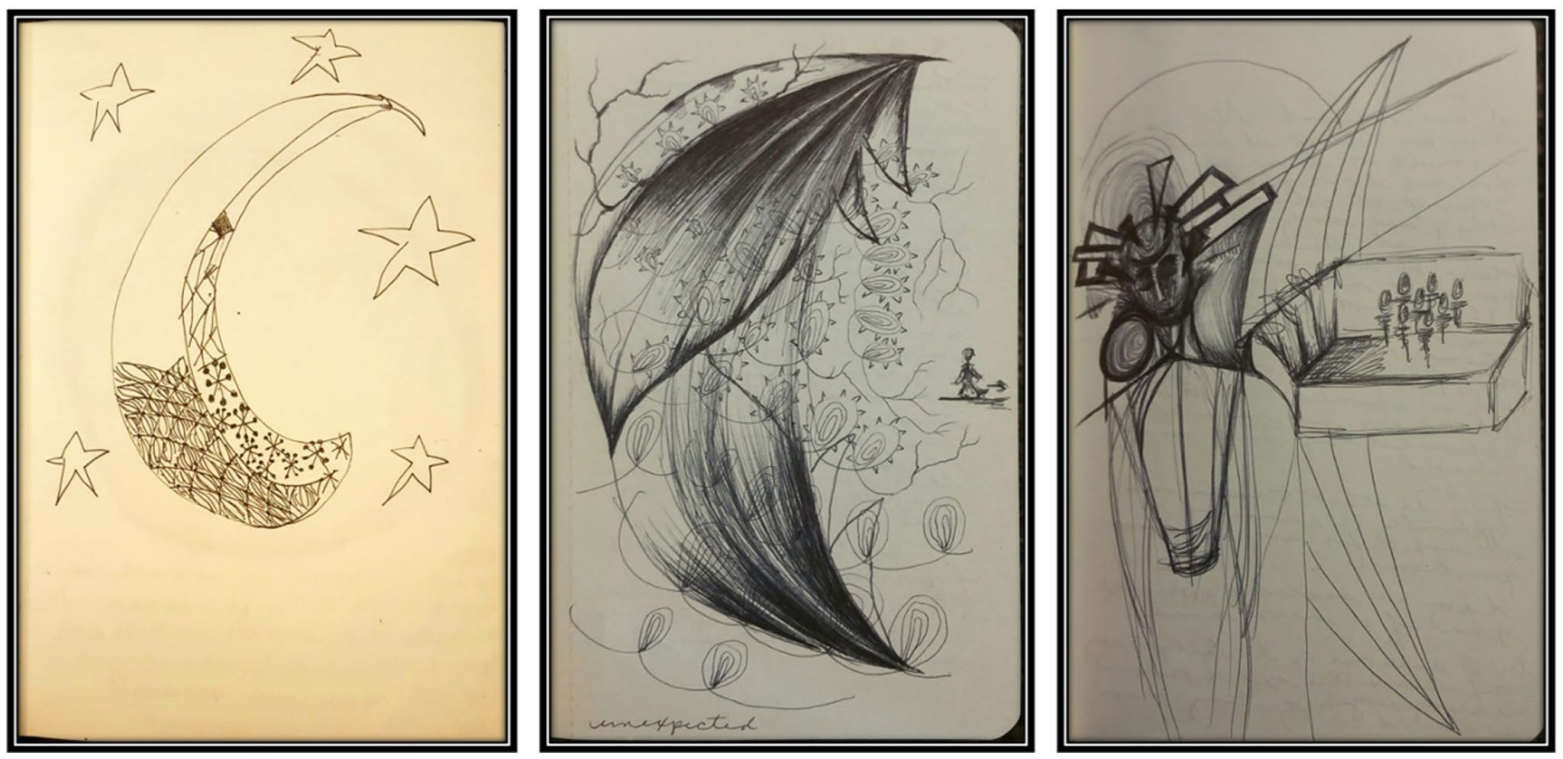
Unprompted child and adult drawings status-post mTBI. Samples of the crescent moon/corpus callosum shape that appeared in child (left) and adult (center and right) spontaneously created imagery following a mTBI. The child’s shape appears like a moon with stars and with some focused incorporation of more concentrated, tighter details along the bottom half of the shape. The adult’s center crescent shape titled *Unexpected* appears ominous with fang-like shapes at the top like it will eat the oblivious figure in its path. The adult’s image on the right shows less concentration on the main crescent shape, but it is the area separating the figure from the group.

When Rheva could manage short visits, Michele visited to bring her an art journal and art supplies. Like Michele, Rheva had been making artwork about *her* mTBI experiences, on her own and unprompted, and shared it at that moment. Since ancient Egypt, illustrations have been a form of medical communication to “draw what cannot be seen” (Perkins, 2024, para. 11). As aforementioned, the CT and MRI scans of TBI patients will look “normal;” not reflecting the reality of pain and discomfort they experience (Lee and Newberg, 2005). While TBI’s invisibility can feel like trickery on part of the person affected, the art does not lie. To the surprise of Michele and Rheva’s mother Denise, we observed commonalities in the spontaneously created art traversing child and adult stages of development. Multiple prevalent themes such as brains, darkened eyes, limp hands, and crescent moons (which we discovered later mimic the shape of the corpus callosum) were too numerous to ignore (Figure 2). The authors’ art therapy with TBI patients further confirmed these common symbol and line formations. From the lens of the art therapist, this compelled us to explore deeper: What were the implications of common symbology in the artwork of mTBI patients across the lifespan?

### A call for interprofessional collaboration for research and practice

As in any art expression created by mTBI individuals, some of the details will connect to the person’s lived experiences of the head injury. However, the common symbols and line quality could demonstrate unconscious characteristics of mTBI artwork of symptoms or experiences that might otherwise elude contemporary neuroimaging. Here, the commonalities in the art linked the child (Rheva) and adult (Michele) despite the two cases being separated by time, age, and origin of injury. The parallel we found most notable was the crescent moon shape, which one neurologist attending one of our conference presentations suggested “looked just like the corpus callosum:” a thick band of nerve fibers connecting the two sides of the brain, responsible for the flow of information between them. Turning the images on their sides, they mimic the C-shape of the corpus callosum; a sensitive area of the mid-brain that can be easily injured by a TBI and not detectable in imaging tests like MRIs (Jang et al., 2019) or functional magnetic resonance imaging (fMRI) (Walker et al., 2018). Symbols have both individual and universal connotations (Granot et al., 2018; Hinz, 2019; Jung, 1968). Like dropping a pebble into a pond and watching the capillary waves extend outward from the peak, our imagined and created inner images can connect with outside images, extending further to a broader, timeless, iconic collective that is a part of being human (Granot et al., 2018). We cannot speculate what exactly a crescent moon would individually mean for each TBI survivor; that is for the individual to define. Looking back on her images, Michele sees fear and pain of being consumed and of being isolated (Figure 2 center and right, respectively). Like the corpus callosum connects the hemispheres, she longed for connection and understanding within herself and amongst her peers and family. Taking a broader, more universal perspective, we can suggest that its existence may indicate an archetypal connection amongst TBI survivors communicating that a part of the brain needs repair. Just because we cannot see the damage does not mean it does not exist.

Typically, if there is no “evidence” of illness or injury, the patient’s description of their experience is misattributed, ignored, or dismissed. When mTBI *is* diagnosed the “mild” classification can be misleading and not accurately predict prognosis (Fromson, 2022). A 2018 study identified a possibility that “visual elements of art therapy products (and their subjective, patient-reported descriptions and meanings) can be correlated with brain activity seen in fMRIs” (Walker et al., 2018, p.185). Although none yet exist, focused neuroimaging and other tests continue to seek the primary brain trauma biomarkers to assist with all levels of mTBI treatment (Schwab et al., 2021; Walker et al., 2018). King et al. (2019) cited recent studies that point toward “assessing the impact of art therapy treatment by measuring changes in client biology” (p. 150). Along these lines, Fromson (2022) echoed Guillamondegui et al. (2011) citing the “suboptimal” long-term and follow up care for TBI patients in the U.S. and stated they would be better served using a 3 to 15 scale of severity known as the Glascow Coma scale, or GSC. This diagnostic tool uses both imagining and bloodwork to define the GSC number. The higher the number the more severe the trauma and the higher the need for care and follow-up. Informed by the work of Walker et al. (2018), art therapy researchers and medical researchers could work together to not only use the GSC classification for more accurate TBI diagnosis, but to parallel it with art therapy products to identify baseline characteristics. There is a need for empirical research that systematically reviews the validity and reliability of such a collaboration, and its implications for effective TBI diagnosis and treatment. At present, there is none that the authors can qualify as evidenced-based, and instead we practice based on the findings of small samples and anecdotal behavioral, qualitative research; not unlike what art therapy clinicians and researchers have identified when developing a neurobiologically-informed framework for the treatment of PTSD (Malhotra et al., 2024). Through an intentional pairing of art therapy products and contemporary medical assessment tools, art therapy could be a graphic map to identify the otherwise elusive symptoms of brain injury, facilitating early intervention and treatment and improving patient-caregiver communication.

The use of arts in medicine is increasingly recognized worldwide (Stuckey and Nobel, 2010) and can add a powerful and therapeutic element to a patient’s healing journey; but this is not therapy. Our recommended collaboration is with credentialed art therapists. They hold a minimum of a Master’s degree, and in the U.S., they are registered and/or board-certified, and in some instances are state-licensed. This is the ideal when utilizing imagery, symbolism, and metaphor for mTBI patient communication and expression, differential diagnostic impressions, and to help the treating provider “see” and understand – from the patient’s perspective –their lived experience. Additionally, mTBI survivors can be a vulnerable population who may be experiencing depression, anxiety, and/or PTSD alongside neurological injury. It is possible that artmaking, media exploration, and the resultant product can be activating; creating the opposite intended effect and triggering a fight or flight response from the amygdala (Hass-Cohen, 2008; Wolf, 2018). “ATR-N [art therapy relational neuroscience] interventions can be designed to take advantage of the neural linkages between ruptured functions of traumatic memories and creativity” (Hass-Cohen, 2025, p. 107). The art therapist can prescribe media and structure interventions that are trauma- and neuroscience-informed (Chapman, 2014), to support and advocate for the patient as needed, or translate (into words) the meaning of their products. For instance, “The location of a stroke or TBI may have differential impacts on a patient’s ability to recognize or respond to emotional cues, or comprehend complex explanations” (Konopka et al., 2024, p. 27). Art products and processes may be helpful in identifying TBI or brain injury as a diagnosis, as well as shaping specific media for use or media quantity in treatment in alignment with the Expressive Therapies Continuum (ETC) and CREATE frameworks. The ETC is a pan theoretical, developmental framework used by art psychotherapists to guide how humans process information and form images (Hinz, 2019; Kagin and Lusebrink, 1978). The six main principles of CREATE are: creative embodiment, relational resonating, expressive communicating, adaptive responding, transformative integration, and empathizing and compassion. They are grounded in the ATR-N approach to guide art therapists in neurobiologically-informed clinical practice, teaching, and research (Hass-Cohen and Findlay, 2019). Both the ETC and CREATE activate cortical and subcortical brain changes, working to integrate different areas of the brain vertically and horizontally, and are useful in memory encoding and retrieval; helping us connect our inner and outer worlds, and make sense of them, with or without words (Hass-Cohen and Findlay, 2019; Van Meter, 2024). Art therapists are ETC- and/or CREATE-educated and have extensive knowledge of developmental imagery and symbol formation across the lifespan, the impacts of biopsychosocial trauma, and corresponding graphic indicators. As clinicians and artists, art therapists provide the entirety of the art therapy continuum, and consultation and evaluation assistance to the treatment team, patient, and their caretakers (see accreditation and professional standards). Additionally, art therapists share the same professional language with the mTBI patient’s treatment team, an international recommendation of current best practices in the treatment of TBI (Rattigan et al., 2021; Wiart et al., 2016).

Considering the complexity of comorbidities of brain injury that may contribute to cognitive challenges, interprofessional, patient-centered treatment should be implemented whenever possible (ASHA, 2013, p.13). Hobson (2006) identified multidisciplinary, interdisciplinary, and transdisciplinary as common team-based approaches in healthcare communities. The WHO (2010) described interprofessional work as “multiple health workers from different professional backgrounds [providing] comprehensive services… working with patients, their families, caregivers, and communities to deliver the highest quality of care across settings” (p. 13). Whatever the treatment structure, we posit that multi-dimensional approaches spanning neurology, psychiatry, and psychology are needed to conceptualize treatment for persistent mTBI symptoms (Clark et al., 2022; Maas et al., 2022), and art therapy is a viable complimentary treatment option from assessment to aftercare. Konopka et al. (2024) contend that “using standardized approaches [such as clinical and objective imaging data, clinical evaluations, etc.] we can move the field of mental health in a very new direction” (p. 32).

## Conclusion

This perspective paper implores readers to take seriously the minimization of mTBI patients’ experiences and how it may impact their care from initial diagnosis and follow-up while understanding that to err is human; it falls in line with our natural human tendency to not believe what we cannot see. We crave empirical evidence yet cannot rely on brain scans and imaging because damage at the cellular level will not appear. From personal and professional experiences, the authors discovered the urgency to create and use imagery to communicate when words failed the mTBI experiencer, or when the words could not accurately depict what they were feeling or sensing. The art became a tool and visual narrative (Berberian et al., 2019; Malhotra et al., 2024) to show others, “*It hurts ‘right’ here*,” or “*It feels just like this*,” in the hopes that the attending physician will finally understand and prescribe the helpful course of treatment. The fact that both cases discussed in this paper and other mTBI patients in the authors’ clinical practices used similar symbols and executed the same line quality regardless of personhood, age, or gender remains an untapped area of research that requires inquiry. The art can and should be taken seriously by the neurological medical community as a supportive and complimentary tool that can assist in developing a treatment plan curated for their patients’ individual mTBI experience. In addition to the work of current art therapist researchers championing this work and bridging neuro-aesthetics, medical art therapy, brain injury rehabilitation via art therapy and biomarkers (Hass-Cohen, 2008, 2025; Hass-Cohen and Findlay, 2019; Kaimal et al., 2017; Kaimal et al., 2016; King et al., 2019; Konopka et al., 2024; Walker et al., 2016; Walker et al., 2018), we are calling for intentional interprofessional collaboration for mTBI research and practice with a focus on commonalities across the lifespan. There is power in art making, and a necessity for credentialed art therapists to provide developmentally appropriate, trauma-informed, culturally responsive, and sensory-aware safety and containment as part of the survivor’s treatment. “The support and skills of an attuned art therapist helps recruit, express and hold the relational self in mind while allowing for the expression of needed emotions and motivations” (Hass-Cohen, 2008, p. 39). The therapeutic relationship, media quality and quantity, directive, placement, and physical space are key. As one of Michele’s patients recalled, they felt validated “on a deep level.” Their art products became graphic maps, like X-rays that mapped their location in the healing process and indicated what areas still required care and attention. Just as there are no imagining tests to “show” mTBI, there are no current imaging modalities that can predict or accurately identify PSC (Rose et al., 2021). Despite ongoing research, presentation of symptoms and recovery rates after TBI remain elusive; approximately half of all hospital patients presenting with mild TBI do not recover to pre-injury wellness after 6 months (Maas et al., 2022). Early treatment of mTBI could reduce PSC onset and the persistence of psychobehavioral symptoms (Goldsmith, 2024; Wiart et al., 2016). As discussed here, the inclusion of art therapy as part of the survivor’s treatment plan can be a welcome addition by both patient and treatment team to help assist with assessment, treatment, and aftercare while also demystifying, naming, and making tangible seemingly idiosyncratic experiences for a more personalized and visible plan of action (Tourgeman et al., 2024).

We leave the reader with an excerpt from a poem written and shared by Dr. John, during the commencement ceremony at Emory University Medical School in 1983, *titled Gaudeamus Igitur* (Therefore, Let Us Rejoice).

For there will be the arts

and some will call them

soft data

whereas in fact they are the hard data

by which our lives are lived

For everyone comes to the arts too late.

For you can be trained to listen only for the oboe

out of the whole orchestra

For you may need to strain to hear the voice of the patient

in the thin reed of his crying (p.1742).

Let us not be too late.

## Data Availability

The original contributions presented in the study are included in the article/supplementary material, further inquiries can be directed to the corresponding author.
