# Corrigendum: Art therapy and brain injury: making the invisible visible

**DOI:** 10.3389/fpsyg.2025.1541074

**Published:** 2025-01-28

**Authors:** Denise R. Wolf, Michele D. Rattigan

**Affiliations:** College of Nursing and Health Professions, Art Therapy and Counseling, Drexel University, Philadelphia, PA, United States

**Keywords:** traumatic brain injury, post-concussive syndrome, art therapy, imagery, expression, interprofessional collaboration, translation neurosciences

In the published article, there was a spelling mistake in the citation details provided for “Malhorta et al., 2024.” Instead of “Malhorta et al., 2024,” it should be “Malhotra et al., 2024.”

The authors apologize for this error and state that this does not change the scientific conclusions of the article in any way. The original article has been updated.
